# Exergetic Assessment for Resources Input and Environmental Emissions by Chinese Industry during 1997–2006

**DOI:** 10.1100/2012/692746

**Published:** 2012-08-22

**Authors:** Bo Zhang, Beihua Peng, Mingchu Liu

**Affiliations:** ^1^School of Management, China University of Mining and Technology (Beijing), Beijing 100083, China; ^2^State Key Laboratory of Coal Resources and Safe Mining, China University of Mining and Technology (Beijing), Beijing 100083, China; ^3^Shenhua International Trading Co. Ltd., Beijing 100011, China; ^4^Pingxiang College, Jiangxi Province, Pingxiang 337000, China

## Abstract

This paper presents an overview of the resources use and environmental impact of the Chinese industry during 1997–2006. For the purpose of this analysis the thermodynamic concept of exergy has been employed both to quantify and aggregate the resources input and the environmental emissions arising from the sector. The resources input and environmental emissions show an increasing trend in this period. Compared with 47568.7 PJ in 1997, resources input in 2006 increased by 75.4% and reached 83437.9 PJ, of which 82.5% came from nonrenewable resources, mainly from coal and other energy minerals. Furthermore, the total exergy of environmental emissions was estimated to be 3499.3 PJ in 2006, 1.7 times of that in 1997, of which 93.4% was from GHG emissions and only 6.6% from “three wastes” emissions. A rapid increment of the nonrenewable resources input and GHG emissions over 2002–2006 can be found, owing to the excessive expansion of resource- and energy-intensive subsectors. Exergy intensities in terms of resource input intensity and environmental emission intensity time-series are also calculated, and the trends are influenced by the macroeconomic situation evidently, particularly by the investment-derived economic development in recent years. Corresponding policy implications to guide a more sustainable industry system are addressed.

## 1. Introduction

The natural resources depletion has been considered as one of the main constraints for sustainable development [1, 2]. Resources, especially nonrenewable resources, are required to supply the basic human needs and to improve the quality of life [3]. At the same time, a majority of nonrenewable resources are consumed in the industry sector, which provides most energy and matter used in modern society. Resources production and consumption by industrial activities are therefore reckoned as a strong positive determinant ingredient of air pollution and climate change [4]. 

In China, the industry sector accounts for approximately 70% of the total energy resources input and consumes the largest amount of mineral resources such as iron ores in the world [5]. Industrial activities along with huge resources input and low resource use efficiency have engendered striking environmental emissions such as greenhouse gases (GHG). Averagely, 81.4% of SO_2_, 80.9% of soot, and 47.8% of waste water in China were emitted by the industry sector during 1997–2006 [6]. In the year of 2006, 24.0 billion ton industrial waste water, 22.3 million ton SO_2_, and 13.0 million ton solid waste were discharged into the environment. The share of CO_2_ emissions from the industry sector accounted for more than two-thirds of China's total energy-related CO_2_ emissions [7]. During a period of rapid economic growth in China, the challenge confronted with the industry sector is the ever-increasing pressure on natural environment due to large amounts of nonrenewable resources consumption with urgent regard for environmental consequences.

Without explicit throughput measures, the scale question of the physical resource base and human conditions cannot be analyzed and reflected adequately [8, 9]. An efficient understanding of the resources use and environmental impact of the Chinese industry against drastic socioeconomic transitions demands systematic biophysical assessment with a unified measure. Exergy is defined using thermodynamics principles as the maximum amount of work which can be produced by a system as it comes to equilibrium with a reference environment [10–12]. The potential usefulness or ability to perform work for a natural resource is its exergy content [13, 14], and then exergy quantifies the quantity and quality scarcities of diverse resources effectively [15]. Distinguishing from the traditional economic analysis, exergy accounting provides a unified way to measure different materials and energy with solid scientific basis [15, 16] and provides a wide and clear vision of the use and degradation of energy and subsequently of natural resources [17, 18]. As an overall and unifying assessing tool, exergy analysis has been widely employed to evaluate the resources use at different scales [19–23], and particularly to perform the resource exergy analysis of different countries [16, 24–40].

Meanwhile, uses of exergy are increasing in fields related to environmental impact. All utilization of resources and disposal of waste products affect nature and the effect is strongly related to the amount of exergy in the utilized resource or the disposed waste [11, 19, 41]. The exergy amount of an emission is the physicochemical work absorbed by the environment in order to equilibrate the substances of the emission with the standard environment [42]. All emissions have definable, calculable, and additive exergy contents with respect to the defined reference environment, and then exergy can be regard as a suitable unifying measure of environmental emissions. Rosen and Dincer [43–45] further stressed that the exergy embodied in waste emissions represents a potential for environmental change. The concept of exergy has been gradually accepted as a “direct” measure or at least as a proxy stated by Ayres [46] for the potential environmental impact of waste emissions [41, 42, 47–50].

Closely relevant to exergy-based insight into resources use and environmental impact of the Chinese industry, Chen and his fellows have carried out a series of studies in their social exergy analysis of resources use and environmental emissions at the national scale covering the industry sector [38–40, 50, 51]: Chen and Qi [38] presented systems account for the resources exergy utilization of China society 2003; G. Q. Chen and B. Chen [39] provided an extend-exergy analysis of the resources conversion and waste emissions of the China society in 2005; Zhang and Chen [40] provided an exergy-based systems account for the resources use and environmental emissions (including GHGs and “three wastes”) of China society 2006; Zhang et al. [50] provided a chemical exergy-based unifying assessment of the “three waste” emissions by Chinese industry during 1997–2006. However, the overall status and trend of the resources use and environmental impact by Chinese industry remain to be revealed systematically with an objective measure to quantify and evaluate various resources and wastes in more aggregated levels.

The aim of this paper is to present an exergetic assessment for the resources input and environmental emissions of the Chinese industry during 1997–2006. By accounting the fundamental utility of resource inflows into Chinese industry including fossil fuels, mineral resources, agricultural and forest products, and other industrial raw materials based on a unified measure, resources use of the Chinese industry is elucidated. Meanwhile, environmental impact of the Chinese industry and in particular, main environmental emissions covering GHGs and “three wastes” are evaluated. Exergy intensities in terms of resource input intensity and environmental emission intensity time-series are also calculated. Corresponding discussion and policy implications coupled with China's macroeconomic situation are presented. In sum, insights provided by exergy analysis in this study can be added to the poor knowledge between industrial economic profitability and ecological sustainability and contribute to resources management and environmental regulation for the policymakers in China.

## 2. Methodology and Data Sources

### 2.1. System Boundary and Data Sources

Chinese industry refers to the material production sector which is engaged in the extraction of natural resources and processing and reprocessing of minerals and agricultural products [6], including (1) extraction of natural resources, such as mining and salt production (excluding hunting and fishing); (2) processing and reprocessing of agricultural products, such as rice husking, flour milling, wine making, oil pressing, silk reeling, spinning and weaving, and leather making; (3) manufacture of industrial products, like steel making, iron smelting, chemicals manufacturing, petroleum processing, machine building, timber processing; water and gas production, and electricity generation and supply; (4) repairing of industrial products such as the repairing of machinery and means of transport (including cars). 

For the national-scale system, the resources input into the Chinese industry contains the imported, gathered, constrained, and extracted commodities as exergy carriers [15, 16]. For avoidance of repetitive and cross calculations, the entrance boundary points are set at the same level of the exergy inflow. Most of relevant environmental resources and economic data for the mainland China are adopted or derived from the official databases and public issued official statistical yearbooks, such as Almanac of China Paper Industry [52], China Environment Yearbook [53], China Food Industry Yearbook [54], China Industrial Economic Statistical Yearbook [55], China Steel Yearbook [56], China Yearbook of Nonferrous Metal Industry [57], and Statistical Yearbook of China [6]. 

### 2.2. Exergy Methodology

In this study, all the thermal exergy of the materials are neglected, for the difference between the temperatures of the materials and the environment is small and therefore the thermal exergy is much less than the chemical exergy of the materials according to the basic definition of exergy [38]. Extensive illustrations for estimating exergy coefficients for different resources in China have been provided by B. Chen and G. Q. Chen [30] and Chen and Qi [38]. Concrete exergy coefficients of the accounted resources are listed in Table 1. 

As to the emission account for the industry system as a macroeconomy, it is reasonable to adopt a global standard environment model to resemble the atmosphere, ocean and earth's upper crust with average geophysical chemical characteristics as the reference environment [60, 61]. The chemical exergy of an emission, as the dominant exergy component is considered in this paper. In China, industrial environmental emissions were not covered in statistics until 1997, and the environmental statistic items only cover the main emissions of the conventional “three wastes.” We extract all the available data for the period from 1997 to 2006 and chose the most remarkable environmental emissions to do a trend analysis. Owing to the data availability, seven major emissions (i.e., CO_2_, CH_4_, COD, SO_2_, soot, dust, and solid waste) are included in our calculations. The emission data of CO_2_ and CH_4_ are taken from Zhang [62] and other emission data from the official published statistical yearbooks [53]. Detailed exergy coefficients of the accounted emissions are presented in Table 2.

## 3. Results

### 3.1. Resources Input

**Figure 1:**
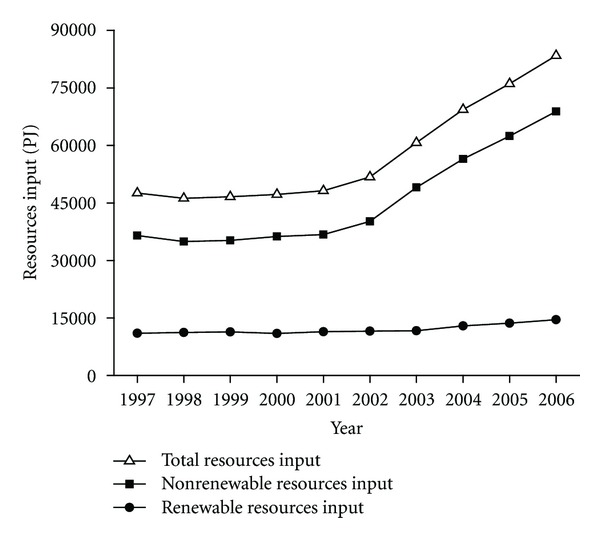
Resources input by Chinese industry.

As the sum of all input fluxes outside the system boundary, a detailed exergy accounting for the resources input of the Chinese industry is performed. Compared with 47568.7 PJ in 1997, resources input in 2006 increased by 75.4% and reached 83437.9 PJ. Concretely, the input of resources exergy kept steady around 46196.5–48187.9 PJ during 1997–2001; while afterwards, it increased from 51777.7 PJ in 2002 to 83437.9 PJ in 2006, with an average annual growth rate of 12.7%. Two categories of resources input are divided, that is, nonrenewable and renewable resources, with corresponding results of exergy accounting shown in Figure 1. The greater part of resource inflows into the industry sector were seen to come from nonrenewable resources, which accounted for 75.6%–82.5% of the total. A rapid increment of the nonrenewable resources input in the recent 5 years can be found, from 40183.3 PJ in 2002 to 68878.6 PJ in 2006, owing to the increasing input of raw coal, crude oil, natural gas, metal and nonmetal minerals into the industrial subsectors. Details are shown in Table 4.

Of all the nonrenewable resources, coal inflow was the largest, contributing to 52.4%–59.9% of the total resources input. In particular, the coal input decreased from 26962.3 PJ in 1997 to 25175.5 PJ in 2001, which can be contributed to rectification and readjustment of coal production performed to balance the wide gap between the supply and demand [16]. During 1998–2001, 58000 small village coal mines were shut down and their production capacity with 2.7 × 10^8^ ton was stopped [63]. Since 2002, the coal production rebounded, restored and continued to increase due to the rapid rise of coal consumption and electricity demand. The input of crude oil also increased 1.9 times in 2006 of that in 1997 and accounted for averagely 18.0% of the total resources input over this period. Natural gas input amounted to 1709.7 PJ, 2.5 times of that in 1997. The iron ore and scrap steel resources input in iron and steel industry increased by 178.7% in the past decade, from 359.5 PJ in 1997 to 1001.9 PJ in 2006. Particularly, the imported iron ore fine and steel product rose rapidly and amounted to 400.0 PJ in 2006, compared with 136.2 PJ in 1997. Nonferrous ores and scrap resources input had increased by more than 3.9 times from 35.4 PJ in 1997 to 136.0 PJ in 2006. As the primary raw material for the cement industry, limestone also expanded 2.4 times during the past ten years. 

**Figure 2:**
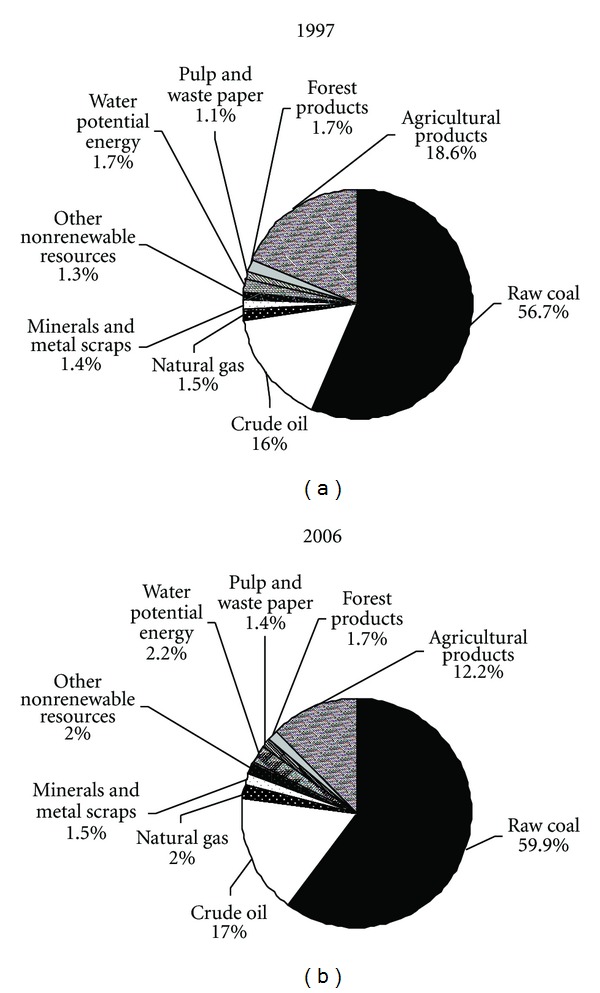
Components of resources input by Chinese industry in 1997 and 2006.

Only a small part of resource inflows from renewable resources, for example, within agriculture and forestry, were used in the industry sector. Renewable resources inflows increased by 31.9% from 11041.4 PJ in 1997 to 14559.2 PJ in 2006, owing to the increasing input of water potential energy, the grains and meats into the food processing industry, and other industrial materials (e.g., wood, pulp, and waste paper). For instance, water potential energy input increased rapidly from 830.0 PJ in 1997 to 1845.7 PJ in 2006, and the imported wood and bean rose by 585.6% and 816.7% in 2006, respectively. Totally, the share of renewable resources in the total resources input decreased from 23.2% (11041.4 PJ) in 1997 to 17.4% (14559.3 PJ) in 2006. Detailed components of resources input by Chinese industry in 1997 and 2006 are shown in Figure 2.

**Figure 3:**
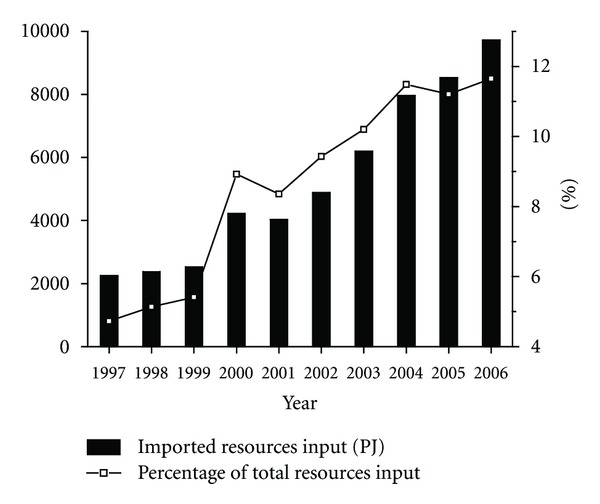
Imported resources input by Chinese industry (Note: Right *y*-axis refers to percentage of total resources input).

Furthermore, the domestic supply of energy and mineral resources always cannot meet the huge and ever-increasing demands in China, and then a large amount of industrial raw materials need to be imported. The total amount of imported resources input into the Chinese industry increased rapidly from 2249.9 PJ (4.7% of the total resources input) in 1997 to 9720.9 PJ in 2006 (11.7% of the total), as shown in Figure 3. As the largest imported resource, crude oil accounted for 67.2% of the total imported resources input for the period between 1997 and 2006 on average.

**Figure 4:**
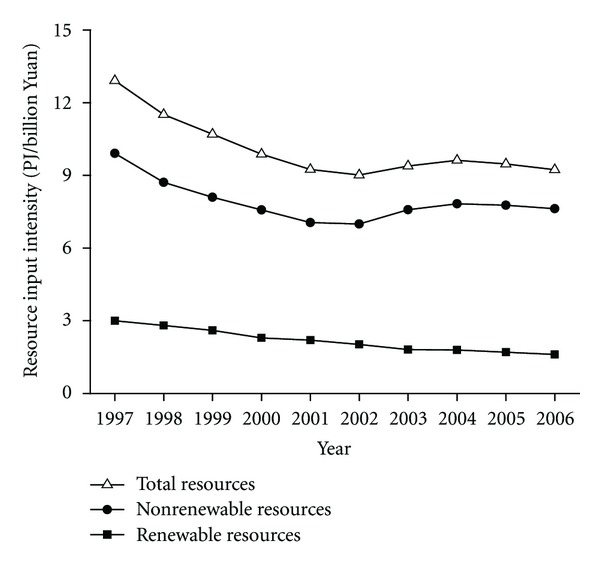
Resource input intensity by Chinese industry.

Resource input intensity (RII), as the ratio of the total exergy input of resources to the total industrial value added (IVA), is a critical parameter for resource policies that aims to reduce resource consumption while maintaining or even boosting economic growth. The lower the ratio, the fewer the resources input to yield per unit IVA and the higher macroeconomic efficiency of resources use in the industrial economy. Macroeconomic output of the Chinese industry along with a large amount of resources input has experienced spectacular uprising with 10.6% average annual growth rate in the total industrial value added (at 2006 constant price, similarly hereafter) over 1997–2006.  Figure 4 presents the resource input intensity of the Chinese industry in this period. The total RII decreased from 12.9 PJ/billion Yuan in 1997 to 9.0 PJ/billion Yuan (1 US$ = 7.7087 RMB Yuan in 2006) in 2002. However, it started to increase by 6.8% over 2003-2004, and declined by 1.6% in 2005 and 4.1% in 2006. As noted previously, the nonrenewable resource input had the dominated share (75.6%–82.5%) in the total resources input. Then the trends of the nonrenewable resource input intensity and the RII show little difference, while the renewable resource input intensity decreased gradually during 1997–2006. Since fossil fuels are the largest resources input, energy intensity measured by the fossil fuels input (including coal, oil, and natural gas) per unit IVA is also calculated. During this period, the energy intensity decreased by 30.2% in 1997–2002, however it rose by 8.1% in 2003 or 11.7% in 2004 and then slightly declined by 2.3% over 2005-2006. 

**Figure 5:**
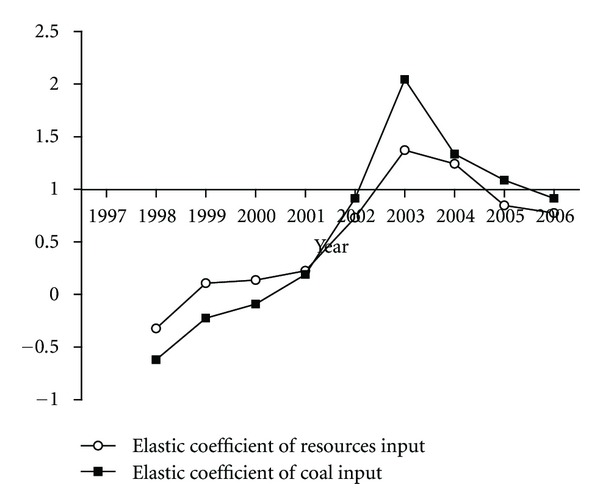
Elastic coefficients of resources input and coal input by Chinese industry.

Displayed in Figure 5 is the resource input elastic coefficient (RIEC) measured by the ratio of the growth rate of resources input to the growth rate of industrial value added [64]. In the detail years, the growth of IVA was faster than the growth of total resources input during 1997–2002 with the average value of the RIEC 0.17; however, the growth of total resources input exceeded the growth of IVA in 2003 and 2004, and the RIEC reached 1.37 in 2003 and 1.24 in 2004; but the RIEC declined to 0.85 in 2005 and 0.77 in 2006. The drastic change of RIEC values is largely due to the change of fossil fuels input. Prominently, the elastic coefficient of resources input changed simultaneously with that of coal input during 1997–2006, as shown in Figure 5. 

### 3.2. Environmental Emissions

**Figure 6:**
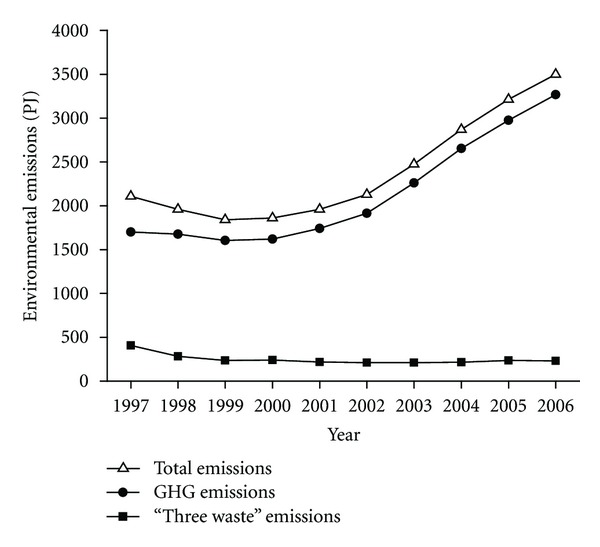
Environmental emissions by Chinese industry.

Environmental emissions can be categorized into GHG emissions and “three wastes” emissions (i.e., waste water, waste gas, and solid waste) in conventional sense. Industrial environmental emissions in terms of GHG emissions and “three wastes” emissions in the past decade (1997–2006) are shown in Figure 6. The total exergy of environmental emissions by Chinese industry amounted to 2107.4 PJ in 1997; however, this figure rose by 66.0% and jumped to 3499.3 PJ in 2006. From the exergetic perspectives, higher exergetic value of the emission reflects the larger deviation in chemical composition from the reference environment and indicates its essential effect on environmental change. In exergy, the GHG emission dwarfs the “three wastes” emission by an order of magnitude and determined the trend of industrial environmental emissions in the whole period to a remarkable extent. In 2006, the total exergy of all the seven primary emissions in 2006 was estimated to be 3499.3 PJ, of which 93.4% was from GHG emissions and only 6.6% from “three wastes” emissions. A rapid growth of the GHG emissions took place for the period between 2002 and 2006, increasing from 1915.7 PJ in 2002 to 3267.2 PJ in 2006 with an average annual growth rate of 14.3%. Meanwhile, the total exergy of “three wastes” emissions did not change remarkably over 1997–2006.

**Figure 7:**
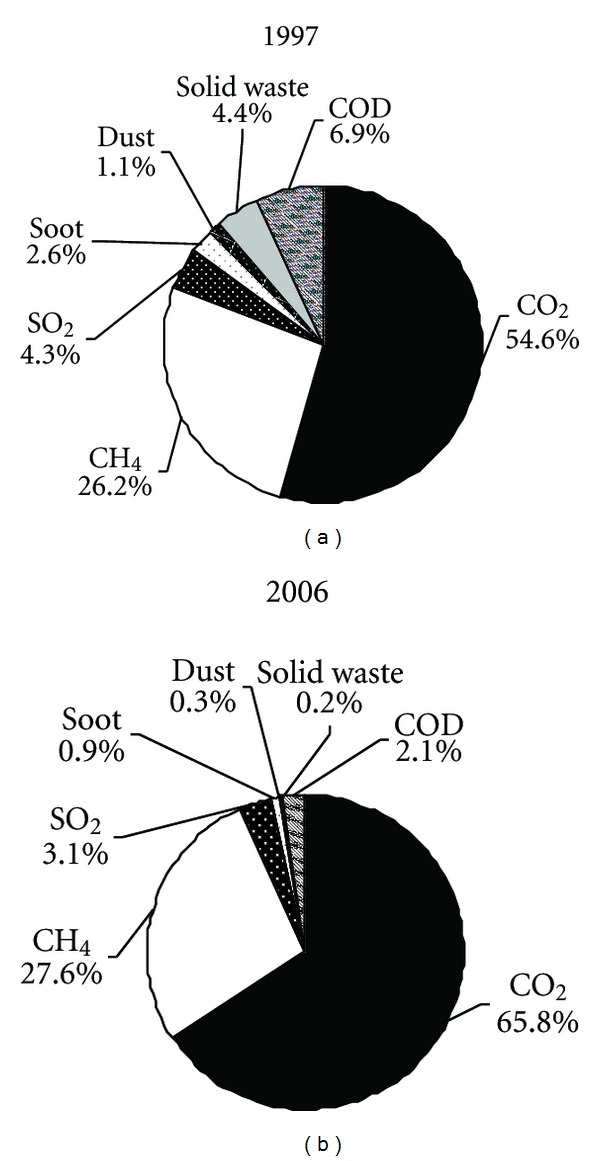
Components of environmental emissions by Chinese industry in 1997 and 2006.

Displayed in Figure 7 is a further comparison of the emission shares in 1997 and 2006. As the largest emission category, the share of the CO_2_ emissions in the total emissions increased from 54.6% (1150.0 PJ) in 1997 to 65.8% (2302.5 PJ) in 2006, followed by the CH_4_ emissions, contributing to 26.2% and 27.6% of the total in 1997 and 2006, respectively. As to the GHG emissions concretely, CO_2_ emissions accounted for 67.6%–74.9% of the total GHG emissions and CH_4_ emissions 30% on average in the past decade. It is worth noting that SO_2_ and COD were the two main pollutants in “three wastes” emissions. The exergy of COD emissions of the Chinese industry decreased from 145.9 PJ in 1997 to 69.3 PJ in 2004, afterward it increased by 8.8% in 2005 and declined by 2.4% in 2006. Meanwhile, SO_2_ emissions decreased by 15.7% in 1997–2002 and then increased rapidly from 76.5 PJ in 2002 to 109.5 PJ in 2006. The emissions of soot, dust, and solid waste experienced a significant drop during 1997–2006. Detailed results of environmental emissions of the Chinese industry during 1997–2006 are shown in Table 5.

**Figure 8:**
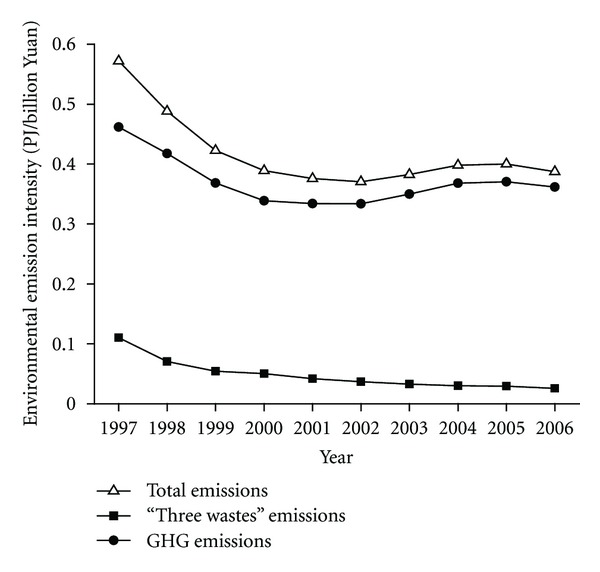
Environmental emission intensity by Chinese industry.

Environmental emission intensity (EEI) defined as the environmental emission exergy per unit of the total industrial value added indicates the environmental effect along with industrial economic output. The lower the EEI, the better environmental performance of industrial activities can be conducted.  Figure 8 displays that the total EEI decreased from 0.57 PJ/billion Yuan in 1997 to 0.37 PJ/billion Yuan in 2002, and then fluctuated slightly during 2002–2006. The GHG emission intensity determined the trend of environmental emission intensity over this period to some extent, increasing its share from 80.7% (0.46 PJ/billion Yuan) in 1997 to 93.4% (0.36 PJ/billion Yuan) in 2006. It is worthy of noting that the time-series trend of the environmental emission intensity is in line with that of the resource input intensity, largely owing to the coal-dominated energy structure in China.

## 4. Discussion

It is worth noting that a majority of the industrial subsectors with high resources input level are the energy-intensive sectors. According to the China Energy Statistical Yearbook [65], the primary end-use energy consumption sectors in industrial system in 2006 were the manufacturing sectors, which accounted for 85.4% of the total industrial energy consumption. Among the manufacturing sectors, the sector of *Smelting and Pressing of Ferrous Metals* made up 25.8% of the total end-use energy consumption, followed by *Manufacture of Raw Chemical Materials and Chemical Products* with 14.9%, and *Manufacture of Nonmetallic Mineral Products* with 12.1% [65]. Correlation analysis shows that the correlation coefficients between mineral resource inflows into the iron and steel industry and energy resource inflows (i.e., coal, petroleum, natural gas) over 1997–2006 were higher than 0.9. Similar results can be found in the nonferrous industry. 

**Figure 9:**
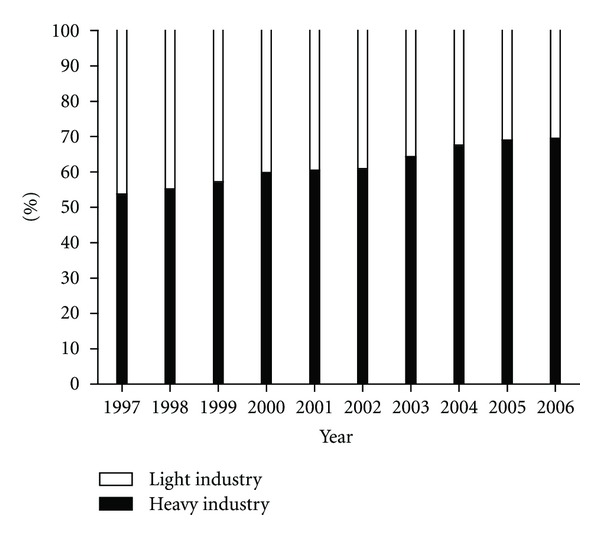
Component of the industrial value added by year.

China is adopting energy-intensive technology and investing the excessive expansion of high-energy consuming sectors, such as iron and steel, cement, and electrolytic aluminum. The outputs of main industrial products, especially most energy-intensive products, increased rapidly during 1997–2006. For instance, the outputs of crude steel, ten major nonferrous metals, motor vehicles, ethylene, cement, plate glass, electricity, chemical fiber, and primary plastic in 2006 were 3.9, 3.3, 4.6, 2.6, 2.4, 2.8, 2.5, 4.4, and 3.8 times of those in 1997, respectively [6, 55]. Some studies in energy intensity (measured by energy consumption with mass units per unit of GDP) reported that the primary driving force for the decline in China's energy intensity during 1997–2002 was efficiency effect rather than sectoral structural shifting [66–68]. It implies, therefore, that technical progress made a notable contribution in the industry during 1997–2002. However, since 2003, the industry sector has raised its production levels and expanded energy-intensive sub-sectors rapidly. Liao et al. [69] also found that the excessive expansion of high-energy consuming sectors and the high investment ratio were foremost sources of the increasing energy intensity during 2003–2005.  Figure 9 further shows that the heavy industry contributed the increasing share to the total industrial value added over 1997–2006 [6]. In 2006, the ratio of the industrial value added of heavy industry to that of light industry reached 70 : 30. 

In fact, the resource utilization level in China still has large gaps in production process, technology, and management, compared with the international advanced level. The average resource extraction efficiency in China is lower than 20%–30% of the global advanced average [70]. As to the production process, the average energy consumption level of equipment and technology in China's manufacturing sectors is more than 10% of that in the OECD countries in general [71]. For instance, the overall energy consumption for per ton of steel, cement, oil refining, ethylene, and calcium carbide output in 2004 were higher than 15.6%, 23.3%, 53.4%, 59.6%, and 19.4% of those in the OCED countries, respectively [72]. The GDP energy intensity in China's industry is also distinctly higher than international levels. According to Yuan et al. [73], the average energy intensity for main products in eight industry sectors of electric power, oil, nonferrous metals, construction material, textile, and others is 40% higher than the world average. Therefore, the potential for promoting resource utilization level is substantial and urgent, especially in some resource-intensive or energy-intensive sectors. At the same time, the industry faces the tremendous challenges of limit resources supply in domestic reserves. It is well known that a large amount of industrial raw materials consumed in China comes from imported goods from the rest of the world. For instance, 50% of the domestic iron ore demand, 33% of alumina, 40% of crude oil, and 44% of wood in 2004 were met through international trade [63]. The pressure for seeking sustained resource supply and improving resource use efficiency is unprecedented.

Furthermore, the rapid growth of materials production and the energy demand for electricity and coal in some major industrial sub-sectors (e.g., steel, electrolytic aluminum, cements, and paper industry) with high-energy consumption and heavy environmental emissions determine the emission profile of the Chinese industry [50, 74–76]. The energy or raw materials utility subsectors are the major sources of industrial environmental emissions. For the period between 1997 and 2006, the sectors of electric power production and coal mining were the leading emitters of CO_2_ and CH_4_ among all the industrial subsectors, respectively [62]. The electric power production, iron and steel production, manufacture of nonmetallic mineral products, nonferrous smelting accounted for about 90% of industrial SO_2_, soot and solid waste emissions in 2004 [77]. It is well known that the quantities of industrial GHG emissions and air pollutants in China are closely related with energy consumption, especially coal consumption [50, 74, 75]. Inefficient and coal-dominated energy production and consumption are at the core of China's environmental emissions. Along with the rapid growth of industrial value added and resources use, the total exergy of industrial “three wastes” emissions has seen a steady decline, though a slight increase of SO_2_ and COD emissions in some years. This effect can be attributed to the effective emission control policies made by the central and local governments. However, GHG emissions of the Chinese industry increased rapidly along with a new rising period of Chinese economy since 2002. It is important to note that China's emissions control programs focus specifically on “three wastes” emissions rather than targeting at greenhouse gases such as carbon dioxide [78]. To tackle the problems of industrial environmental emissions, a more international way of thinking instead of a regional approach should be taken, with specially emphasis on the greenhouse gases rather than the regional pollutants merely [50].

Prominently, the resources use and environmental impact of the Chinese industry have been notably influenced by the macroeconomic situation in the last decade.  Table 3 presents the three components of GDP by expenditure approach during 1997–2006. Totally, final consumption expenditure and gross capital formation shared the majority proportion of the GDP over this period. During 1998–2001, the economic growth was largely derived by the domestic demand. After 2002, the situation started to overturn: the contribution of gross capital formation in China's total GDP exceeded that of the final consumption expenditure. Investment has become an important motor for China's economic growth in recent years [76, 79]. Most of the investment flows into manufacturing, infrastructure, and real estate related sectors [80], which enormously pushes up the demand for certain resource- and energy-intensive products, such as steel, nonferrous metals, cement, glass, and machine. Since the second half year of 2003, the government had implemented a series measures to strengthen macro-control, with specially emphasis on the control of the investment in fixed assets, land supply management and environmental regulation [63]. There was significant decline in the growth rate of investment and total investment for new planned projects, especially heavy industry investment after 2005, while slight decline of resource input intensity and environmental emission intensity by Chinese industry over 2005-2006 can be found.

## 5. Concluding Remarks

Natural resources from the ecological system are required for producing and supplying goods and service in the industry system. Environmental emission assimilation as an additional ecological input into the industry sector can also be regarded as the use of an “ecological service.” For sustainable development, natural resources, especially nonrenewable resources should not run out and environmental emissions should not endanger the ecological system [40]. Given China's rapid industrial expansion, policy-makers require a more detailed understanding of the complex linkages between industrial activities and natural environment if the resultant resource use and environmental impact are to be minimized. In this paper, an exergy-based physical assessment is performed to measure the resources use and environmental impact of the Chinese industry for the period between 1997 and 2006.

The resources input into the Chinese industry reached 83437.9 PJ in 2006, and increased by 75.4% compared with that in 1997. For the time-series trend, resources input showed little variation during 1997–2001 and the initial trend had changed since 2002 with the input levels showing a great rebound. Nonrenewable resources accounted for 75.6%–82.5% of the total and determined the trend of resources input to a certain extent. A rapid increment of the nonrenewable resources input in the recent 5 years can be found, from 40183.3 PJ in 2002 to 68878.6 PJ in 2006 with an average annual growth rate of 14.5%. Coal input was the largest contributor, accounting for 52.4%–59.9% of the total resources input during the period, followed by crude oil and natural gas. The imported resources input increased its share from 4.7% of the total resources input (2249.9 PJ) in 1997 to 11.7% in 2006 (9720.9 PJ), mainly coming from crude oil import. 

The environmental emissions by Chinese industry increased from 2107.4 PJ in 1997 to 3499.3 PJ in 2006. In exergy, the GHG emission dwarfs the “three wastes” emission by an order of magnitude and determined the trend of industrial environmental emissions in the whole period to a remarkable extent. The total exergy of all the seven primary emissions in 2006 amounted to 3499.3 PJ, of which 93.4% was from GHG emissions and only 6.6% from “three wastes” emissions. A rapid growth of total GHG emissions took place for the period between 2002 and 2006, increasing from 1915.7 PJ in 2002 to 3267.2 PJ in 2006 with an average annual growth rate of 14.3%. As the largest emission category, the CO_2_ emissions increased its share from 54.6% of the total emissions in 1997 to 65.8% in 2006, followed by CH_4_ emissions contributing averagely 26% to the total. The exergy of “three wastes” emissions did not change remarkably over 1997–2006, and SO_2_ and COD were the two main pollutants. 

Exergy intensities in terms of resource input intensity and environmental emission intensity time-series are calculated. The resource input intensity declined for the period between 1997 and 2002, but it started to increase over 2003-2004 and then declined slightly in 2005 and 2006. The environmental emission intensity in the whole period shows a similar trend. Moreover, the development of macroeconomic efficiencies of resources input and environmental emissions can be split into two main periods with different characteristics: the first period from 1997 to 2001 corresponding to a more notable improvement in resource and environmental efficiency; the second period from 2002 onwards with faster increased nonrenewable resources input into resource—or energy-intensive subsectors under slower yield of industrial value added. The excessive expansion of high-energy consuming industrial subsectors and the high investment ratio in the macroeconomic structure were foremost sources of the increasing exergy intensities. To obtain the industrial value added of one billion Yuan (129 million US$) in 2006, the resources input and environmental emissions by Chinese industry were estimated to be 9.2 and 0.4 PJ, respectively.

Industry plays an important role in Chinese economy. The contributions of industrial value added to the increase of the GDP in China reached 47.0%–58.3% and the shares in the GDP were around 40% during 1997–2006 [6], which means that China relied on manufacturing industry to an unusually great extent. Nevertheless, skyrocketing resources input and environmental emissions of the Chinese industry mean a surging and huge pressure into the ecosystem. The depletion of the resources brings on the economic increase, and the resulting wastes are returned to the environment where they induce environmental pollution and climate change. Also the development of the Chinese industry can hardly become more resource and energy intensive that it is now, along with the limited resource reserves and adverse environmental quality. Therefore, increasing GDP based on traditional industrialization mode on the expense of natural environment is unsustainable. Continued strong emphasis on sustainability requires that future industrial economic growth must rely much more on environmental friendly and be less dependent on material products and natural resources than in the past. A large effort has to be made to promote industrial structure adjustment, strength resources management and resource efficiency improvement, rationalize resource prices, and implement more stringent energy saving and emission control policies. More importantly, the policies of restructuring and transformation of the resource-intensive economic growth pattern in China will affect and improve the whole situation of resources use and environmental impact of the industry sector.

## Figures and Tables

**Table 1:** Exergy coefficient of various resources.

Item	Exergy coefficient	Unit	Source
Fossil fuels			
Coal	22.16	PJ/Mton	[38]
Oil and oil product	44.32	PJ/Mton	[38]
Natural gas	4.13	PJ/10^8^ cu·m	[38]
Minerals			
Iron ore (55% Fe)	0.46	PJ/Mton	[38]
Iron ore fine (70% Fe)	0.84	PJ/Mton	[38]
Sulphur iron ore (35% S)	9	PJ/Mton	[38]
Copper ore (0.65% Cu)	0.03	PJ/Mton	[30]
Lead ore (3.5% Pb)	0.02	PJ/Mton	[30]
Zinc ore (5.9% Zn)	0.05	PJ/Mton	[30]
Copper ore fine (23.8% Cu)	1.1	PJ/Mton	[38]
Alumina (63.7% Al)	2	PJ/Mton	[38]
Phosphorite (25% P_2_O_5_)	0.1	PJ/Mton	[38]
Raw salt (NaCl)	0.2	PJ/Mton	[38]
Limestone	0.01	PJ/Mton	[30]
Metal scraps			
Steel (Fe)	6.8	PJ/Mton	[38]
Copper (Cu)	2.1	PJ/Mton	[38]
Aluminum (Al)	32.9	PJ/Mton	[38]
Forest products			
Wood	10	PJ/Mton	[30]
Bamboo	18.67	PJ/Mton	[58]
Turpentine	37.4	PJ/Mton	Calculated by authors
Oil-tea camellia seed	35.3	PJ/Mton	Calculated by authors
Tung oil	38.9	PJ/Mton	Calculated by authors
Agricultural products			
Sugarcane	5	PJ/Mton	[38]
Cotton	16.4	PJ/Mton	[30]
Hemp	16.35	PJ/Mton	[30]
Rapeseed	37	PJ/Mton	[30]
Beet	5	PJ/Mton	[30]
Soybean	3.9	PJ/Mton	[30]
Cocoon	4.5	PJ/Mton	[30]
Wool	3.7	PJ/Mton	[30]
Peanut	24.6	PJ/Mton	[30]
Sesame	23.4	PJ/Mton	[30]
Tubers	3.7	PJ/Mton	[30]
Bean	3.9	PJ/Mton	[30]
Rice	15.56	PJ/Mton	[30]
Wheat	15.4	PJ/Mton	[30]
Corn	8.6	PJ/Mton	[30]
Tobacco leaf	10.7	PJ/Mton	[30]
Pork	25	PJ/Mton	[30]
Beef	11.5	PJ/Mton	[30]
Mutton	16	PJ/Mton	[30]
Poultry	4.5	PJ/Mton	[30]
Milk	4.9	PJ/Mton	[30]
Egg	6.2	PJ/Mton	[30]
Fruit	1.9	PJ/Mton	[30]
Aquatic product	5.77	PJ/Mton	[30]
Straw	14.3	PJ/Mton	[30]
Other raw materials			
Pulp	17	PJ/Mton	[30]
Rubber	32.48	PJ/Mton	[30]
Synthetic rubber	45.53	PJ/Mton	[59]
Ethylenc glycol	19.34	PJ/Mton	[60]
Terephthalic acid	24.8	PJ/Mton	[60]
Polyethylene in primary forms	48.26	PJ/Mton	[60]
Polypropylene in primary forms	47.7	PJ/Mton	[59]
Polystyrene in primary forms	50.2	PJ/Mton	[60]
Polyvinyl chloride in primary forms	20.35	PJ/Mton	[59]

Note: The exergy coefficients of water potential energy and nuclear energy were deduced from their product of electricity (0.36 PJ/10^8^ kWh) with the transformation factor of 1.17 and 3.51, respectively [38]. Some chemical materials, nonmetallic mineral, and other raw material are not included due to their negligible exergy input or scarcity data.

**Table 2:** Exergy coefficient of various emissions.

Item	Exergy coefficient	Unit	Source
CO_2_	0.45	PJ/Mton	[60]
CH_4_	51.98	PJ/Mton	[60]
COD	13.6	PJ/Mton	[40]
SO_2_	4.9	PJ/Mton	[60]
Soot	3.5	PJ/Mton	[50]
Dust	1.5	PJ/Mton	[50]
Solid waste	0.5	PJ/Mton	[50]

**Table 3:** The three components of GDP by expenditure approach.

Year	Final consumption expenditure		Gross capital formation		Net exports of goods and services	
	Share	Contribution to the GDP growth	Share	Contribution to the GDP growth	Share	Contribution to the GDP growth
	(%)	(percentage points)	(%)	(percentage points)	(%)	(percentage points)
1997	37.0	3.4	18.6	1.7	44.4	4.2
1998	57.1	4.4	26.4	2.1	16.5	1.3
1999	74.7	5.7	23.7	1.8	1.6	0.1
2000	65.1	5.5	22.4	1.9	12.5	1.0
2001	50.0	4.1	50.1	4.2	−0.1	0
2002	43.6	4.0	48.8	4.4	7.6	0.7
2003	35.3	3.5	63.7	6.4	1.0	0.1
2004	38.7	3.9	55.3	5.6	6.0	0.6
2005	38.2	4.0	37.7	3.9	24.1	2.5
2006	39.2	4.3	41.3	4.6	19.5	2.2

Data source: [6].

**Table 4:** Resources input by Chinese industry, 1997–2006 (Unit: PJ).

Resource category	1997	1998	1999	2000	2001	2002	2003	2004	2005	2006
Nonrenewable resources	36527.3	34953.4	35259.0	36264.4	36956.1	40183.3	49028.7	56466.1	62427.3	68878.6
Raw coal	26962.3	25473.4	24987.0	24759.4	25175.5	27521.6	34606.9	39917.9	44861.6	49979.4
Crude oil	7622.1	7633.0	8321.2	9330.3	9381.8	9908.8	10977.4	12686.8	13278.0	14218.5
Natural gas	697.6	708.3	744.2	834.3	899.5	939.6	1106.0	1212.6	1461.2	1709.7
Iron ores and scraps	359.5	344.1	337.1	359.7	411.3	466.7	572.4	703.4	855.1	1001.9
Nuclear energy	182.2	178.1	188.8	211.5	220.7	317.3	547.5	637.5	670.6	692.7
Nonferrous metal ores and scraps	35.4	32.3	34.9	57.0	47.1	66.8	79.2	106.3	128.3	136.2
Nonmetal minerals	247.8	128.7	121.9	101.6	95.8	100.9	97.3	136.9	127.4	136.0
Other nonrenewable resources	420.5	455.6	523.8	610.8	724.4	861.6	1042.0	1064.8	1045.1	1004.1
Renewable resources	11041.4	11243.2	11353.2	10984.2	11231.9	11594.5	11695.2	12928.6	13659.0	14559.3
Water potential energy	830.0	880.9	863.2	941.9	1174.9	1219.8	1201.6	1497.2	1681.5	1845.7
Pulp and waste paper	535.2	536.9	589.7	596.6	619.1	718.6	808.7	907.4	1022.8	1168.0
Forest products	831.5	835.1	798.9	795.0	818.0	924.8	1037.5	1124.3	1207.2	1382.2
Agricultural products	8844.6	8990.2	9101.3	8650.7	8620.0	8731.4	8647.5	9399.8	9747.5	10163.4

Total	47568.7	46196.6	46612.2	47248.6	48187.9	51777.7	60723.9	69394.7	76086.3	83437.9

Imported resources	2249.9	2374.5	2521.9	4216.6	4027.8	4882.7	6192.2	7967.1	8525.7	9720.9

**Table 5:** Environmental emissions by Chinese industry, 1997–2006 (PJ).

Emission category	1997	1998	1999	2000	2001	2002	2003	2004	2005	2006
GHGs	1701.3	1676.0	1604.6	1620.8	1740.7	1915.7	2261.7	2653.5	2976.0	3267.2
CO_2_	1150.0	1169.6	1178.5	1213.4	1259.5	1349.3	1582.3	1837.2	2077.9	2302.5
CH_4_	551.4	506.4	426.1	407.3	481.2	566.4	679.4	816.4	898.2	964.7
Three wastes	406.1	283.1	236.0	240.5	218.2	211.4	212.0	215.4	236.9	232.0
SO_2_	90.7	78.1	71.5	79.0	76.8	76.5	87.8	92.7	106.3	109.5
Soot	54.8	41.1	33.4	33.4	29.4	28.1	29.6	31.0	33.2	30.3
Dust	22.6	19.8	17.6	16.4	14.9	14.1	15.3	13.6	13.7	12.1
Solid waste	92.1	35.2	19.4	15.9	14.5	13.2	9.7	8.8	8.3	6.5
COD	145.9	108.9	94.1	95.8	82.6	79.4	69.6	69.3	75.5	73.6

Total	2107.4	1959.2	1840.6	1861.2	1958.8	2127.1	2473.8	2868.9	3212.9	3499.3

## Appendix

See Tables 4 and 5.
